# Inhibition of Human Neutrophil Extracellular Trap (NET) Production by Propofol and Lipid Emulsion

**DOI:** 10.3389/fphar.2019.00323

**Published:** 2019-04-05

**Authors:** Angela Meier, Jason Chien, Laura Hobohm, Kathryn Ann Patras, Victor Nizet, Ross Corriden

**Affiliations:** ^1^Department of Pediatrics, Division of Host-Microbe Systems and Therapeutics, University of California, San Diego, La Jolla, CA, United States; ^2^Institut für Physiologische Chemie, Stiftung Tierärztliche Hochschule Hannover, Hanover, Germany; ^3^Department of Pharmacology, University of California, San Diego, La Jolla, CA, United States

**Keywords:** neutrophil, neutrophil activation, inflammation, sepsis, bacteria

## Abstract

Uncontrolled bacteremia is a common and life threatening condition that can lead to sepsis and septic shock with significant morbidity and mortality. Neutrophil granulocytes, the most abundant phagocytic leukocyte of the innate immune system, play an essential role in capturing and killing invading pathogens. Their antimicrobial repertoire includes the formation of Neutrophil Extracellular Traps (NETs), chromatin-based, web-like structures of DNA that facilitate the capture and killing of bacteria. In sepsis, however, it has been suggested that the uncontrolled release of NETs worsens disseminated coagulation and promotes venous thrombosis. Here, we describe how clinically relevant concentrations of the commonly used sedative propofol as well as a lipid composition similar to the propofol carrier impair NET production by human neutrophils. Drugs commonly administered in the Intensive Care Unit (ICU) may impact the inflammatory response to either worsen or improve clinical outcomes and may therefore be considered for additional therapeutic effects if clinical studies confirm such findings.

## Introduction

Uncontrolled inflammation leading to a dysregulated immune response is a hallmark of sepsis and can impair organ system function, damage host tissues and render the host unable to resolve infections (Boomer et al., 2011; Hotchkiss et al., 2013). As the most abundant circulating white blood cells, neutrophils are key drivers of such effects (Dohrmann et al., 2016). Neutrophil Extracellular Traps (NETs), chromatin-based antimicrobial structures (Brinkmann et al., 2004) that are released from neutrophils in response to both exogenous and endogenous stimuli, have been implicated as key drivers of such effects. Although NETs facilitate the capture and killing of microbes, thus limiting their dissemination from the initial tissue nidus of infection, uncontrolled NET production in sepsis has been linked to hypercoagulability, microvascular dysfunction (McDonald et al., 2017), and increased risk of thromboembolism (Yang et al., 2017). Indeed, NETs have recently been linked to worsened outcomes in severe influenza infections (Zhu et al., 2018), and extracellular histones [among the most abundant proteins in NETs (Brinkmann et al., 2004)] have been described as contributors to sepsis mortality (Xu et al., 2009). Thus, while the bloodstream enables the rapid deployment of circulating neutrophils to new infectious foci throughout the body, hyperactivation of the NET phenotype within the bloodstream may represent a “dark side” of neutrophils (Sorensen and Borregaard, 2016) in late-stage infection.

During sepsis or septic shock, patients often require aggressive supportive care in the Intensive Care Unit (ICU) and may receive sedative drugs to tolerate such support. One of the most widely used agents in the ICU setting is propofol, an intravenous anesthetic agent diluted in a lipid emulsion with rapid onset of action after infusion or bolus injection paired with a very short recovery period. Propofol positively modulates the inhibitory function of the neurotransmitter γ-aminobutyric acid (GABA) via GABA_A_ receptors (Trapani et al., 2000). Although propofol has been shown to have anti-inflammatory effects (summarized in Meier and Nizet, 2018), the effects of propofol on neutrophil function are not fully understood. In particular, the effect of clinical propofol formulations on NET production has not previously been reported. In this study, we aimed to examine the impact of clinically relevant concentrations of injectable propofol and its carrier/vehicle lipid emulsion on the ability of human neutrophils to produce NETs, and the impact of these agents on neutrophil antimicrobial function against the leading ICU bacterial pathogen, methicillin-resistant *Staphylococcus aureus* (MRSA).

## Methods

### Neutrophil Isolation

The UC San Diego Human Research Protection Program (HRPP) Institutional Review Board (IRB) approved the described studies (IRB# 131002). Under informed consent, male and female healthy human subjects were phlebotomized, and neutrophils were isolated from blood using 1 Step Polymorphs^TM^ (Fresenius Kabi Norge AS) per the manufacturer’s instructions. Cells were resuspended in Phosphate Buffered Saline (PBS) to 1 × 10^7^ cells/ml then further diluted in media described below depending on the assay performed. Cells were incubated for the indicated times with propofol (Hospira or Aztec, Inc., containing per ml: 10 mg of propofol, 100 mg of soybean oil, 22.5 mg Glycerin, 12 mg egg lecithin, 1.5 mg benzyl alcohol, 0.7 mg sodium benzoate, and sodium hydroxide) or lipid emulsion [Nutrilipid 20% IV Fat emulsion (Braun), containing per ml: 200 mg soybean oil, 25 mg glycerin, egg phospholipids USP 12 mg, and sodium oleate 0.3 mg]. The concentration of propofol used in the described experiments was 5 μg/ml. The concentration of lipid emulsion (Nutrilipid) was adjusted to be equivalent to propofol based on the most prevalent component of the carrier solution (soybean oil).

### NET Quantification

Neutrophils were incubated for 30 min with propofol at a concentration of 5 μg/ml or a corresponding concentration of lipid emulsion (adjusted to soybean oil content). Cells were then treated with either 25 nM PMA for 4 h or nigericin for 3 h at 37°C with 5% CO_2_, followed by addition of micrococcal nuclease (1 U/mL; Dako Denmark). After an additional 10-min incubation at 37°C, enzymatic activity was stopped via addition of 10 mM EDTA. Cells were then centrifuged for 5 min at 200 ×*g*, and extracellular DNA in supernatant samples quantified using the Quant-iT PICO Green^TM^ dsDNA Assay Kit (Invitrogen) and an EnSpire Alpha Multimode Plate Reader (PerkinElmer).

### NET Visualization

Neutrophils were incubated with either propofol or lipid emulsion for 30 min at 37°C with 5% CO_2_ prior to addition of 25 nM phorbol 12-myristate 13-acetate (PMA; Sigma). Following an additional 2-h incubation at 37°C with 5% CO_2_, cells were fixed via addition of paraformaldehyde (4% final concentration). Fixed neutrophils were blocked with PBS plus 2% bovine serum albumin and goat serum, followed by staining using an anti-human myeloperoxidase antibody (1:300; Dako Denmark) and an Alexa 488-labeled secondary antibody (1:500; Life Technologies) as previously described (Corriden et al., 2015). Cells were imaged using an AxioObserver D1 Inverted Fluorescence Microscope (Zeiss) equipped with an LD A-Plan 20X/0.35 Ph1 objective and processed with affinity photo (Serif).

### Reactive Oxygen Species (ROS) Quantification

Neutrophils (2 × 10^6^ cells/ml) were incubated for 20 min at 37°C with gentle agitation in Ca^2+^/Mg^2+^-free Hanks’ Balanced Salt Solution (HBSS) with 10 μM 2’,7’-dichlorodihydrofluorescein diacetate (DCFDA; Thermo Fisher Scientific). Following a 5-min centrifugation at 1200 rpm, cells were resuspended in HBSS with Ca^2+^/Mg^2+^ at a density of 2 × 10^6^ cells/ml with or without propofol or lipid emulsion. After a 30-min incubation at 37°C, cells were seeded in 96-well plates at a density of 2 × 10^5^ cells/well. PMA was added to applicable wells, and ROS generation quantified using an EnSpire Alpha Multimode Plate Reader (PerkinElmer), with reads collected every 15 min for 2 h (485 nm excitation, 530 nm emission). Between reads, plates were incubated and protected from light at 37°C with 5% CO_2_. To account for variability between donors and dye loading between experiments, data were expressed as percentages of the maximum fluorescence intensity detected within each experimental repeat.

### Bacterial Killing Assays

Human neutrophils were pre-incubated with propofol (5 μg/mL), lipid emulsion or vehicle (RPMI with 2% FBS) alone for 30 min. MRSA (USA300 strain LAC) was grown from overnight cultures to an optical density (600 nm) of 0.4 (log phase), resuspended in RPMI with 2% FBS, added to neutrophils at a multiplicity of infection (MOI) of 10:1 (bacteria to neutrophil), and incubated for 45 min at 37°C and 5% CO_2_. Subsequently, samples from the neutrophil/bacteria co-cultures were serially diluted in water and triplicate samples plated onto Todd Hewitt Agar (THA) for enumeration of bacterial colony forming units (CFU) after overnight incubation at 37°C.

### Statistical Analysis

The significance of acquired data was tested statistically using GraphPad Prism 7 software. For experiments in which multiple groups were tested, One Way Analysis of Variance (ANOVA) with *post hoc* Dunnett’s Multiple Comparisons test was used to assess statistical significance. For all experiments, listed replicates (i.e., “*n*”) represent the number of independent experiments performed, each using neutrophils isolated from unique donors.

## Results

### Clinically Relevant (Khan et al., 2014) Concentrations of Propofol and Corresponding Concentrations of Lipid Emulsion Significantly Inhibit NET Production

**FIGURE 1:**
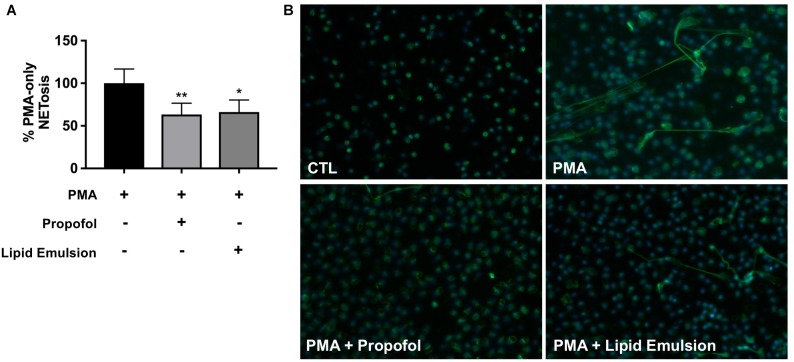
Propofol and lipid emulsion inhibit PMA-induced NET production. **(A)** PMA-induced NET production/extracellular DNA in the presence or absence of propofol/lipid emulsion was quantified using the fluorescent DNA stain PicoGreen (*n* = 4). Data were analyzed using one-way ANOVA (*p* = 0.0033) with Dunnett’s multiple comparisons test (*p* = 0.0086 PMA vs. PMA + propofol; *p* = 0.0153 PMA vs. PMA + lipid emulsion). **(B)** PMA-induced NET production with and without pre-treatment with propofol or lipid emulsion was visualized via fixation of neutrophils and staining using an antibody against myeloperoxidase (*n* = 3; representative image shown). Propofol concentration was 5 μg/mL for all experiments, with lipid emulsion concentration adjusted to match the soybean oil concentration in propofol.

Plasma concentrations of propofol following IV administration have previously been reported to fall within a range of 2–30 μg/ml (Khan et al., 2014). NET production, induced by the Protein Kinase C (PKC) activator phorbol 12-myristate 13-acetate (PMA), was quantified in the presence or absence of propofol (5 μg/ml) via fluorescence-based measurement of extracellular DNA (Figure 1A) and fluorescence microscopy (Figure 1B). Because propofol is only available for injection into patients in a lipid formulation, we assessed in parallel the effect of lipid emulsion (adjusted to be roughly equivalent to the propofol solution according to the concentration of the major component soybean oil). Inhibition of NET production was observed in response to both propofol and lipid emulsion treatment. It has previously been reported that neutrophils do not express GABA_A_ receptors, the primary pharmacological target of propofol; thus, our results suggest that the observed effects of propofol are largely driven by the lipid carrier.

### Propofol and Lipid Emulsion Inhibit PMA-Induced ROS Production

**FIGURE 2:**
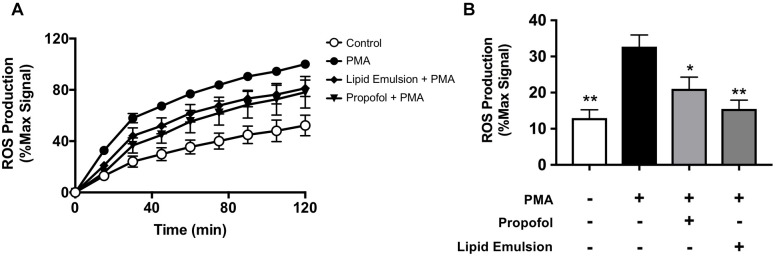
Propofol and lipid emulsion inhibit PMA-induced ROS production. ROS production in untreated (control) and PMA-treated cells with and without propofol or lipid emulsion was assessed using the fluorescent ROS probe DCFDA over time (**A**; *n* = 3) and at 15 min-post PMA addition (**B**; *n* = 3). Data were analyzed using one-way ANOVA (*p* = 0.0049) with Dunnett’s multiple comparisons test (*p* = 0.003 PMA vs. CTL; *p* = 0.0474 PMA vs. PMA + propofol; *p* = 0.0066 PMA vs. PMA + lipid emulsion). Propofol concentration was 5 μg/mL for all experiments, with lipid emulsion concentration adjusted to match the soybean oil concentration in propofol.

PMA-induced NETosis is known to be ROS-dependent (Kenny et al., 2017). Given that both propofol and lipid emulsion inhibited PMA-induced NET production, we sought to determine whether these formulations also altered PMA-induced ROS production. Using the membrane permeable fluorescent ROS probe DCFDA, we found that both propofol and lipid emulsion inhibit PMA-induced ROS production, suggesting a possible mechanism underlying inhibition of NETosis (Figure 2A,B).

### Neither Propofol nor Lipid Emulsion Affect ROS-Independent NET Production

**FIGURE 3:**
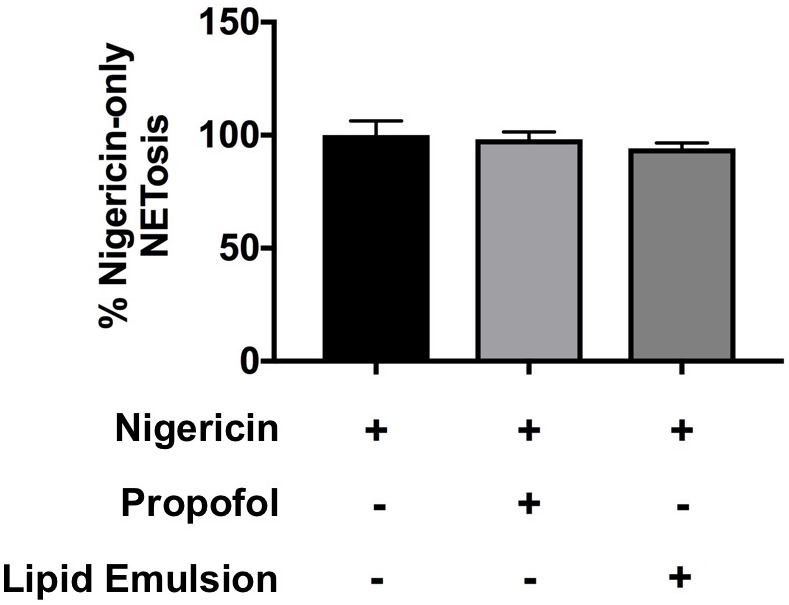
Neither propofol nor lipid emulsion alter NETosis in response to the ROS-independent NET inducer nigericin. The effect of propofol and lipid emulsion on nigericin-induced NETosis was quantified using the PicoGreen method (*n* = 3). No statistical difference in NET production was observed between groups. Data were analyzed using one-way ANOVA with Dunnett’s multiple comparisons test. Propofol concentration was 5 μg/mL for all experiments, with lipid emulsion concentration adjusted to match the soybean oil concentration in propofol.

Because NETs can be produced through both ROS-dependent and ROS-independent pathways (Kenny et al., 2017), we also assessed the impact of propofol and lipid emulsion on NET production induced via a ROS-independent pathway using nigericin derived from *Streptomyces hygroscopicus*, finding that nigericin-induced NETs were not significantly altered by either agent (Figure 3; *p* = 0.88 ctl (nigericin alone) vs. nigericin+propofol; *p* = 0.66 ctl (nigericin alone) vs. nigericin+lipid emulsion).

### Neither Propofol nor Lipid Emulsion Affect Growth or Neutrophil Killing of USA300 Methicillin-Resistant *S. aureus* (MRSA)

**FIGURE 4:**
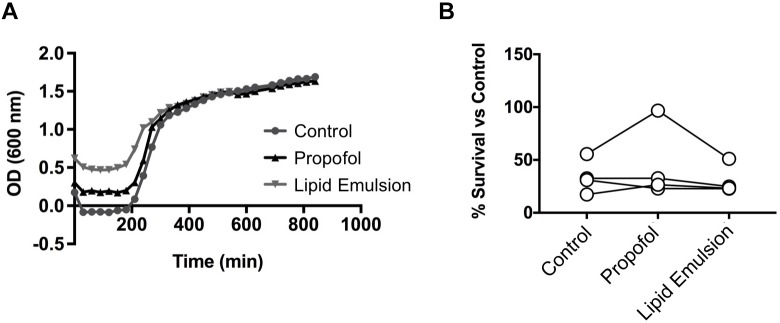
Neither propofol nor lipid emulsion affect growth or neutrophil killing of USA300 MRSA. **(A)** USA300 MRSA growth in the presence or absence of propofol or lipid emulsion was quantified by measuring absorbance of cultures at OD_600_ (*n* = 3). **(B)** Killing of USA300 MRSA by neutrophils following a 45-min co-culture (MOI 10) was quantified via plating of co-culture samples and enumeration of bacterial colonies (*n* = 4). Data were analyzed using one-way ANOVA with Dunnett’s multiple comparisons test. Propofol concentration was 5 μg/mL for all experiments, with lipid emulsion concentration adjusted to match the soybean oil concentration in propofol.

Because NETs are an important antimicrobial defense mechanism of human neutrophils, we determined whether propofol and lipid emulsion interfered with the overall capacity of neutrophils to kill a common ICU bacterial pathogen, MRSA. Neither propofol nor lipid emulsion interfered significantly with neutrophil killing of MRSA (Figure 4B; *p* = 0.58 ctl vs. propofol; *p* = 0.51 ctl vs. lipid emulsion). Furthermore, neither propofol nor lipid emulsion had any direct effect on MRSA growth, as determined via growth-curve analysis (Figure 4A).

## Discussion

Our findings described in this brief report show that clinically relevent concentrations of propofol and lipid emulsion significantly inhibit ROS-dependent NET production by human neutrophils. However, the overall ability of human neutrophils to effectively kill MRSA was not impaired by propofol or lipid emulsion, and neither propofol nor lipid emulsion impacted bacterial growth. The inclusion of lipid emulsion, used in our experiments as a carrier control for propofol, is of particular interest as neutrophils reportedly do not express GABA_A_, the receptor known to directly interact with propofol (Sanders et al., 2015). Our results suggest that the inhibitory effects of clinical propofol preparations on ROS-dependent NET production could potentially be mediated by the lipid components of the drug, as corroborated in independent studies of lipid emulsions on neutrophil ROS production (Heine et al., 1999). Because propofol is only available for clinical application in a lipid emulsion and because our carrier control, nutrilipid, a lipid emulsion similar to the propofol carrier, is also used to meet caloric needs in the ICU, our results may be of importance in critical care beyond sedative use only.

The balance between effective bacterial elimination and detrimental dysregulated over-activation of the immune system has been a topic of substantial investigation and has been suggested to be impacted by drugs commonly administered during critical illness such as norepinephrine (Stolk et al., 2016). While our studies did not demonstrate an impact of propofol or lipid emulsion on human neutrophil killing of MRSA, we did demonstrate a significant impact of clinically relevant concentrations of propofol on the production of NETs, which in sepsis are shown to be associated with unfavorable outcomes (Yost et al., 2009; Czaikoski et al., 2016; McDonald et al., 2017; Yang et al., 2017).

It is of crucial importance to understand how sedation during sepsis impacts the inflammatory response as our most critically ill patients are exposed to sedation for prolonged periods of time. Further studies in humans, such as clinical trials examining inflammatory markers and incidence of venous thrombosis (VT) and venous thromboembolism (VTE) in sepsis controlling for different anesthetics and sedatives used, are warranted to determine clinical significance of the described studies.

## Author Contributions

AM and RC planned and performed the experiments, analyzed the data, generated the figures, and wrote the manuscript. JC and LH performed experiments. KP and VN provided input into experimental planning and edited the manuscript.

## Conflict of Interest Statement

The authors declare that the research was conducted in the absence of any commercial or financial relationships that could be construed as a potential conflict of interest.
